# Identification of the First Gene Transfer Agent (GTA) Small Terminase in *Rhodobacter capsulatus* and Its Role in GTA Production and Packaging of DNA

**DOI:** 10.1128/JVI.01328-19

**Published:** 2019-11-13

**Authors:** D. Sherlock, J. X. Leong, P. C. M. Fogg

**Affiliations:** a Biology Department, University of York, York, United Kingdom; University of Illinois at Urbana Champaign

**Keywords:** horizontal gene transfer, bacterial evolution, gene transfer agent, *Rhodobacter*, antimicrobial resistance, terminase, DNA packaging, bacteriophage, transduction, virus, bacterial evolution, DNA-binding proteins, antibiotic resistance, bacteriophage assembly, gene transfer

## Abstract

Genetic exchange mediated by viruses of bacteria (bacteriophages) is the primary driver of rapid bacterial evolution. The priority of viruses is usually to propagate themselves. Most bacteriophages use the small terminase protein to identify their own genome and direct its inclusion into phage capsids. Gene transfer agents (GTAs) are descended from bacteriophages, but they instead package fragments of the entire bacterial genome without preference for their own genes. GTAs do not selectively target specific DNA, and no GTA small terminases are known. Here, we identified the small terminase from the model Rhodobacter capsulatus GTA, which then allowed prediction of analogues in other species. We examined the role of the small terminase in GTA production and propose a structural basis for random DNA packaging.

**IMPORTANCE** Random transfer of any and all genes between bacteria could be influential in the spread of virulence or antimicrobial resistance genes. Discovery of the true prevalence of GTAs in sequenced genomes is hampered by their apparent similarity to bacteriophages. Our data allowed the prediction of small terminases in diverse GTA producer species, and defining the characteristics of a “GTA-type” terminase could be an important step toward novel GTA identification. Importantly, the GTA small terminase shares many features with its phage counterpart. We propose that the GTA terminase complex could become a streamlined model system to answer fundamental questions about double-stranded DNA (dsDNA) packaging by viruses that have not been forthcoming to date.

## Abstract (precis)

Random transfer of any and all genes between bacteria could be influential in the spread of virulence or antimicrobial resistance genes. Discovery of the true prevalence of GTAs in sequenced genomes is hampered by their apparent similarity to bacteriophages. Our data allowed the prediction of small terminases in diverse GTA producer species, and defining the characteristics of a “GTA-type” terminase could be an important step toward novel GTA identification. Importantly, the GTA small terminase shares many features with its phage counterpart. We propose that the GTA terminase complex could become a streamlined model system to answer fundamental questions about double-stranded DNA (dsDNA) packaging by viruses that have not been forthcoming to date.

## INTRODUCTION

Viral transduction by bacteriophages is generally accepted to be the dominant mechanism for the rapid exchange of genes between bacteria. Viruses are the most abundant organisms in the environment; it is estimated that there are >10^30^ viruses in the oceans alone, and the majority of these are viruses of bacteria (1). The impact of bacteriophages is massive, from their crucial role in biogeochemical cycling in the oceans to the ubiquitous crAss phages that are intimately associated with >98% of tested human gut microbiomes (2, 3).

True viruses are essentially selfish—they use host resources to replicate their own genome and package it into the viral protein shell before the progeny move on to infect a new host. Host DNA can also be packaged by bacteriophages, but this occurrence is usually incidental (4–6). In contrast, gene transfer agents (GTAs) are small virus-like particles that exclusively package and transfer random fragments of their host bacterium’s DNA to recipient bacteria (7, 8), with no preference for the propagation of their own genes. There are no known restrictions on the DNA that can be packaged into GTA particles, and consequently, any gene may be transferred by GTAs (8–10). An eye-opening study of antibiotic gene transfer by GTAs in *in situ* marine microcosms detected extraordinary transfer frequencies that were orders of magnitude greater than more established mechanisms (11).

GTAs were first discovered in the alphaproteobacterium Rhodobacter capsulatus, which remains the model organism for the study of GTAs today (12, 13). The R. capsulatus GTA (RcGTA) is contained in a 14.5-kb core gene cluster that encodes a phage T4-like large terminase and most of the RcGTA structural proteins (portal, capsid, various tail proteins, and glycoside hydrolases) required for RcGTA production (14). Recently, ectopic loci encoding tail fibers, head spikes, and putative maturation proteins have also been identified (15, 16). Homologous clusters of RcGTA-like genes are present throughout the *Alphaproteobacteria* and appear to have coevolved with the host species, indicative of vertical inheritance (17, 18). Beyond the *Alphaproteobacteria*, functional GTAs have since been discovered experimentally in diverse prokaryotes, including animal pathogens of the *Brachyspira* genus (spirochete) (19), *Desulfovibrio* spp. (deltaproteobacteria) (20), and the archaeon Methanococcus voltae (21, 22). Each of these disparate GTAs was identified by chance during the study of phage-like particles or unusual levels of gene transfer. However, it is extremely difficult to systematically identify GTAs by bioinformatics alone, because they are functionally analogous but genetically divergent from each other and their genes strongly resemble remnant bacteriophages. The difficulty of rapidly identifying GTAs is perhaps the major obstacle for expanding the breadth of research carried out on GTA producers.

The packaging of random bacterial DNA by GTAs is a fundamentally different behavior from that of bacteriophages and other viruses (23). The primary aim of a phage is to distribute its own genes. Phages first replicate their genome, usually as a multicopy concatemer. There is no evidence that GTAs possess any DNA replication genes or that the packaged DNA has been replicated; instead, GTAs appear to contain the uncopied genome of the producing bacterium. For viruses, in all known cases the volume of the capsid is enough to contain the whole viral genome; however, this is not the case for GTAs (7). An individual GTA virion is too small to package the genes required for its own synthesis; for example, each RcGTA transfers only ∼4 kb of DNA, but the 14.5-kb core gene cluster plus several ectopic loci are required for mature GTA production. To achieve packaging specificity, double-stranded DNA (dsDNA) phages usually use initiation sites at a specific location in the phage genome that are recognized by the packaging machinery. Packaging initiation sites generate specificity with a defined DNA sequence, e.g., *cos*/*pac* sites (23–25), or with favorable topological features, e.g., conformational selection of intrinsically bent DNA by SPP1-like phages (26). So far, no evidence that GTAs target discrete packaging start sites has been presented and no conserved sequences or topologies have been implicated as *cos/pac* equivalents, all of which suggests that packaging initiation is indeed random.

Bacteriophages with a dsDNA genome use sophisticated molecular machinery, known as the terminase, to specifically recognize replicated phage DNA and to drive it into a preformed capsid (27). The capsid itself is essentially a passive receptacle, and it is the terminase that provides DNA selectivity, enzymatic activity, and motive force required to fill the capsid. The terminase is a complex of two oligomeric small and large terminase proteins, TerS and TerL, which are both indispensable for proper phage function and DNA packaging (27). TerL possesses the enzymatic activities required for DNA packaging: it has a C-terminal nuclease domain that cleaves the target DNA to produce a free end available for packaging and an N-terminal ATPase domain that translocates the DNA into a preformed capsid. Unlike TerL, TerS has no enzymatic activity and instead carries out a regulatory role, being responsible for recognition of the phage genome’s packaging initiation site, recruitment of TerL, and modulation of TerL enzymatic activities (26, 28, 29).

In general, large terminase genes are sufficiently well conserved to allow confident identification by sequence identity alone, partly owing to the presence of the Walker ATP-interacting motifs, and thus most GTAs have an annotated *terL* gene. Small terminases, however, are smaller than large terminase genes, with little primary sequence conservation, which makes them far more challenging to identify *in silico*. No small terminase has been identified for any GTA to date. Given the role of terminase proteins in phage biology, it is highly likely that the GTA terminase plays a defining role in the packaging of random DNA. In this study, we definitively identify the small terminase of the model R. capsulatus GTA, demonstrate and localize its interaction with the large terminase, and investigate its role in RcGTA production. Our characterization of the RcGTA TerS also allows us to speculate on the physical requirements of a GTA-type small terminase and to identify candidate small terminases in other GTA-producing species.

## RESULTS

### Characterization of RcGTA *g1* (*rcc01682*).

**FIG 1:**
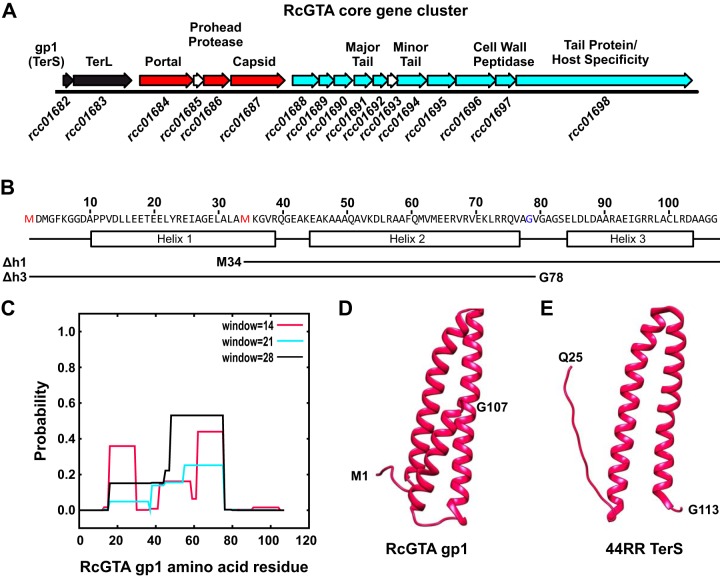
Location and structure of the RcGTA small terminase, gp1. (A) Schematic of the RcGTA core gene cluster. Genes are shown as arrows, with R. capsulatus SB1003 gene designations indicated below and known or predicted protein functions indicated above. Arrows are colored according to type: DNA packaging, black; head associated, red; tail associated, cyan. (B) Amino acid sequence of RcGTA gp1 with the predicted secondary structure indicated. Boxes represent α-helices, and lines are disordered. The boundaries of helix 1 (Δh1) and helix 3 (Δh3) truncations used in this study are shown as lines beneath the sequence, with the new terminal amino acids annotated. (C) RcGTA gp1 coiled-coil prediction using COILS. The three window sizes for the prediction are annotated on the graph and color coded. (D) 3D structural prediction of RcGTA gp1 using RaptorX and visualized using UCSF Chimera. Terminal amino acids are annotated. (E) Crystal structure of *Aeromonas* phage 44RR TerS, visualized with UCSF Chimera.

**FIG 2:**
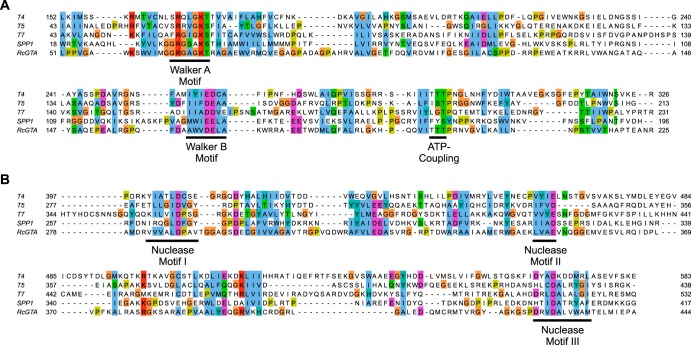
Conserved functional motifs of the RcGTA large terminase protein, gp2. Alignments of the N-terminal ATPase domains (A) and C-terminal nuclease domains (B) of RcGTA gp2 (GenBank accession no. ADE85428), phage T4 gp17 (AAD42422), phage T5 TerL (AAS77194), phage T7 gp19 (AAP33962), and phage SPP1 gp2 (CAA39537). Alignments were made using COBALT and visualized using Jalview. Amino acid similarity is indicated using the Clustal color scheme. Amino acid position numbers in the full-length protein are shown at the beginning and end of each row. The location of the Walker A, Walker B, motif III/ATP-coupling, and nuclease motifs are underlined and annotated (30).

A gene encoding a TerL homologue (*rcc01683/*RcGTA *g2*) is readily identifiable within the Rhodobacter capsulatus SB1003 core RcGTA gene cluster (Fig. 1A). The RcGTA TerL has regions of strong homology with large terminases from several well-studied phages, including the presence of characteristic nuclease and Walker ATPase motifs (Fig. 2) (30, 31). Small terminases are far more difficult to predict, and consequently no GTA small terminases have ever been identified. Most characterized phage TerS proteins have a modular structure: the N-terminal region comprises the helix-turn-helix DNA-binding domain, the central region contains a coiled-coil oligomerization domain, and the C terminus contains the TerL interaction segment (32). Such domain organization is a well-conserved feature of TerS, despite the lack of sequence conservation.

In phage genomes, the small and large terminase genes are often colocalized, and so the core RcGTA gene cluster was examined for genes that could encode a small terminase. The RcGTA gene cluster contains 17 predicted genes, of which at least 6 have been shown to be essential for GTA production (16). The first gene of the cluster, *rcc01682* (referred to hereafter as *g1* and the protein as gp1), is also thought to be essential for RcGTA activity (33), but no in-depth characterization has been carried out and no function has so far been assigned. The *g1* open reading frame (ORF) is 324 bases and is located immediately upstream of the large terminase. The gp1 protein sequence was submitted to the JPRED4 protein secondary-structure prediction server (34), which predicted an almost entirely helical structure (Fig. 1B). Subsequent analysis using the COILS server (35) (MTIDK matrix, all window sizes) indicated that of the three distinct α-helices, the first two are likely to form a coiled coil (Fig. 1C) reminiscent of a phage TerS oligomerization domain. A more detailed structural prediction using the RaptorX structure prediction server (36) indicated similarity to the phage T4-like small terminase from *Aeromonas* phage 44RR (Fig. 1D and E). The 44RR TerS crystal structure (PDB ID 3TXS) failed to resolve the N- and C-terminal segments (residues 1 to 24 and 114 to 154, respectively) due to conformational variability; however, an N-terminal helix-turn-helix DNA-binding motif was predicted from the primary sequence (32). RcGTA gp1 begins with the coiled-coil domain and appears to lack a DNA-binding domain (Fig. 1D) at the N terminus. No helix-turn-helix motif was detected by the Gym2.0 and NPS@ servers (37–39).

**FIG 3:**
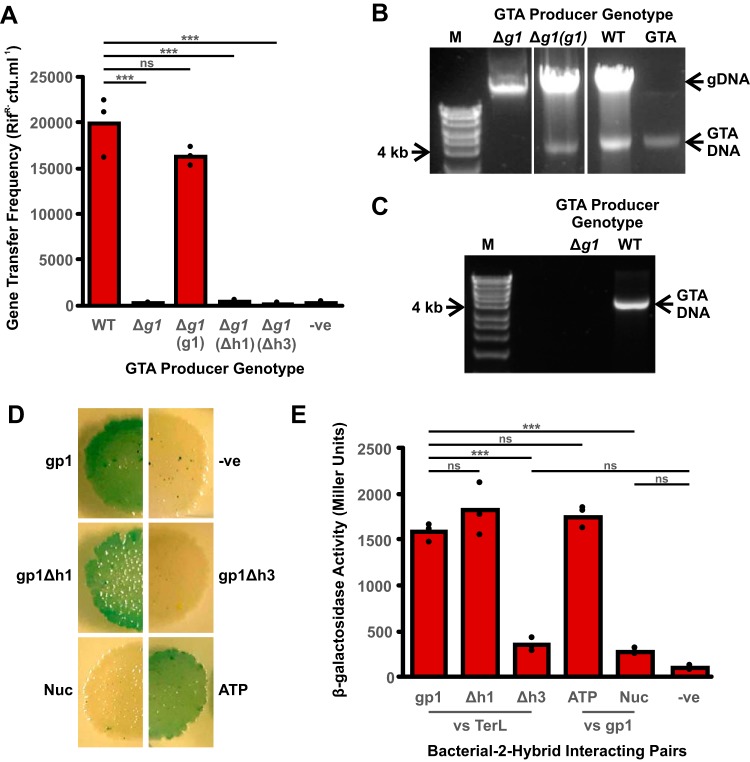
Role of gp1 in RcGTA production. (A**)** Histogram showing the results of a gene transfer assay using the following donor strains: R. capsulatus DE442 wild type (WT), *g1* deletion strain (Δ*g1*), *g1* deletion strain complemented with full-length *g1* [Δ*g1*(*g1*)], *g1* deletion strain complemented with *g1* lacking helix 1 [Δ*g1*(Δh1)], and *g1* deletion strain complemented with *g1* lacking helix 3 [Δ*g1*(Δh3)]. Statistical significance is shown above the chart (ANOVA, *n* = 3; ***, *P* < 0.01; ns, not significant). (B) Agarose gel of total DNA isolated from R. capsulatus DE442 wild type (WT), *g1* deletion strain (Δ*g1*), and a complemented *g1* deletion strain [Δ*g1*(*g1*)]. Bioline HyperLadder 1kb DNA ladder (M) and purified RcGTA DNA (GTA) are shown for size comparison. The locations of genomic DNA, RcGTA DNA, and the 4-kb DNA ladder band are annotated. (C) Agarose gel of DNA isolated from purified RcGTA particles released by DE442 wild-type and *g1* deletion strains. (D) Interaction between RcGTA gp1 and the large terminase (TerL). Ten-microliter spots are the results of individual bacterial two-hybrid assay transformations. Blue/green indicates a positive interaction, and white indicates no interaction. Reactions shown are as follows: “gp1,” gp1 versus TerL; “-ve,” no insert control; “gp1Δh1,” gp1 helix 1 deletion versus TerL; “gp1Δh3,” gp1 helix 3 deletion versus TerL; “Nuc,” gp1 versus TerL nuclease domain; “ATP,” gp1 versus TerL ATPase domain. (E) Histogram showing quantification of the interactions shown in panel D by a β-galactosidase assay. Statistical significance is shown above the chart (ANOVA, *n* = 3; ***, *P* < 0.01; ns, not significant).

To confirm that *g1* is essential for RcGTA activity, a deletion mutant was produced in the GTA hyperproducer strain R. capsulatus DE442. Loss of the *g1* gene prevented all detectable gene transfer activity (Fig. 3A). Complementation with full-length *g1* expressed ectopically from its own promoter effectively restored gene transfer to wild-type (WT) frequencies (Fig. 3A). Complementation was also attempted using *g1* constructs that lacked the sequence encoding either the first or third α-helical regions; in both cases, gene transfer frequencies were indistinguishable from those of the uncomplemented DE442 Δ*g1* mutant (Fig. 3A).

It has previously been shown that the DE442 RcGTA hyperproducer packages sufficient genomic DNA into GTA particles to allow detection of a distinct 4-kb band in total DNA preparations (40). Given the predicted headful packaging mechanism used by RcGTA (8, 27), production of 4-kb DNA fragments can only occur if DNA is successfully packaged into the capsid. This property can be exploited to examine factors that affect DNA packaging *in vivo*, independent of the release of infective GTA particles. Deletion of *g1* prevents any detectable accumulation of intracellular RcGTA 4-kb DNA fragments, and in *trans* complementation restores DNA packaging (Fig. 3B). DNA contained within extracellular GTA particles is protected from enzymatic degradation. Isolation of DNase-insensitive DNA from the supernatant of DE442 wild-type and Δ*g1* strains yielded detectable RcGTA DNA for the wild-type only (Fig. 3C). As phage TerS proteins are responsible for binding to target DNA and stimulating the various enzymatic activities of the TerL proteins that are required for DNA packaging, our data are entirely consistent with *g1* encoding the RcGTA small terminase.

### The C terminus of RcGTA gp1 interacts with the ATPase domain of TerL.

In bacteriophage, the only protein that the small terminase is known to interact with is the large terminase. Indeed, the small terminase not only recognizes the bacteriophage DNA but also recruits the large terminase and initiates the process of DNA packaging. Using the bacterial two-hybrid assay, RcGTA gp1 was translationally coupled to the T25 domain of the Bordetella pertussis adenylate cyclase enzyme and RcGTA TerL was coupled to the adenylate cyclase T18 domain. Interaction between the two proteins brings together the two adenylate cyclase domains, leading to cAMP production and subsequently β-galactosidase (41). In this assay, a distinct interaction can be seen between gp1 and TerL (Fig. 3D). Truncation of gp1 to remove helix 1 had no appreciable effect on the interaction with the large terminase; however, loss of helix 3 led to a complete loss of interaction (Fig. 3D). Quantification of the results with a colorimetric β-galactosidase assay showed no significant difference between the helix 3 deletion and the no-insert negative control (analysis of variance [ANOVA], *n* = 3, *P* = 0.248), whereas the helix 1 deletion result was indistinguishable from that for full-length gp1 (ANOVA, *n* = 3, *P* = 0.253) (Fig. 3E).

The RcGTA large terminase has clear homology with large terminase proteins of several well-studied phages (Fig. 2). The N terminus of the protein contains the ATPase domain with conserved Walker A and B motifs (Fig. 2A), while the C terminus contains the nuclease domain, including three conserved nuclease motifs (Fig. 2B) (30). In the well-studied T4-like phages, it is the ATPase domain that directly interacts with the small terminase (42). To test whether the ATPase domain of the RcGTA large terminase is also responsible for interaction with gp1, translational fusions were made of each of the two domains with the adenylate cyclase T18 domain. In a bacterial two-hybrid assay, the TerL nuclease domain (V253-L455) did not interact with RcGTA gp1 but the ATPase domain (L27-V258) produced a signal indistinguishable from that of full-length TerL (ANOVA, *n* = 3, *P* = 0.480) (Fig. 3D and E).

### RcGTA gp1 production is a prerequisite for tail attachment and efficient GTA capsid maturation.

**FIG 4:**
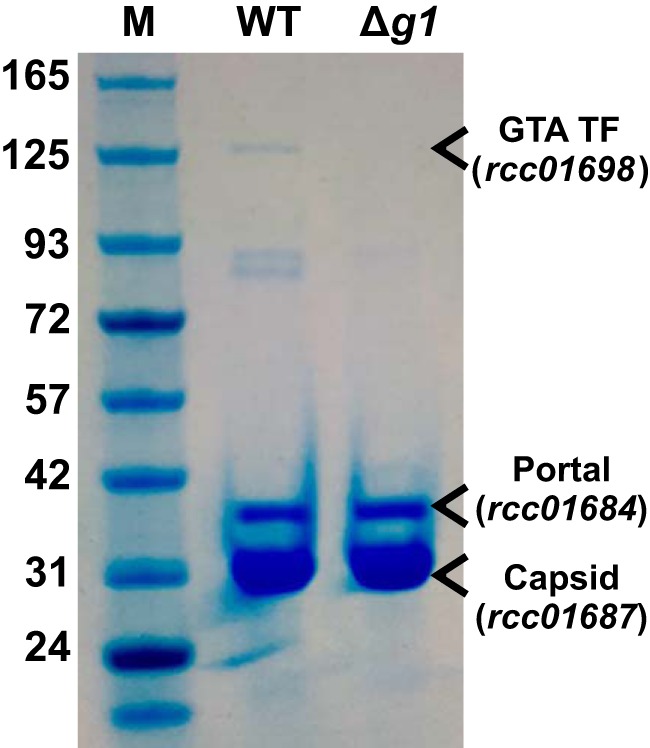
Comparison of the major structural proteins in wild-type RcGTA and a gp1 knockout. SDS-PAGE gel of affinity-purified RcGTAs produced by DE442 wild-type (WT) and *g1* deletion (Δg1) strains. The Expedeon tricolor marker is included for size comparison (lane M), with approximate molecular weights (in kilodaltons) indicated to the left of the gel. Bands predicted to contain the RcGTA portal and capsid proteins are annotated, as well the tail fiber (GTA TF) that was confirmed by MALDI-mass spectrometry.

As shown above, Δ*g1* mutants are unable to produce infective GTA particles or to package DNA, which indicates that RcGTA production has stalled early in the assembly process. To determine the developmental state of the stalled RcGTAs, we purified the RcGTA particles that were released by DE442 WT and Δ*g1* strains during lysis by using nickel affinity purification. The RcGTA lysis genes (*rcc00555* and *rcc00556*) are located elsewhere in the R. capsulatus genome and should not be affected by the absence of a small terminase (8, 43). A plasmid containing the RcGTA capsid (*rcc01687*/RcGTA *g5*) with a C-terminal His_6_ tag was introduced into WT DE442 and isogenic Δ*g1* strains. The timing of capsid expression was matched to GTA production by fusing the *g5* ORF directly to the previously characterized RcGTA promoter (40, 44). Incorporation of recombinant capsid monomers into nascent RcGTA particles allows affinity purification of the whole particles, as previously described (15). Concentrated samples were run on an SDS-PAGE gel to qualitatively assess the relative protein content. Strong bands were evident in both samples at sizes consistent with the RcGTA capsid (posttranslationally processed to 31.4-kDa [45]) and portal (42.8-kDa) proteins (Fig. 4). RcGTA^WT^, but not RcGTA*^g1^*, also had several other visible bands (Fig. 4). RcGTA particles contain a distinctive 138.9-kDa putative tail fiber/host specificity protein (encoded by *rcc01698*/RcGTA *g15*) (45), and a band of this size was present only in the RcGTA^WT^ lane (Fig. 4). The band was excised and positively identified as gp15 by matrix-assisted laser desorption ionization-tandem mass spectrometry (MALDI-MS/MS) (three unique peptide hits; expect < 0.05; total score, 154).

**FIG 5:**
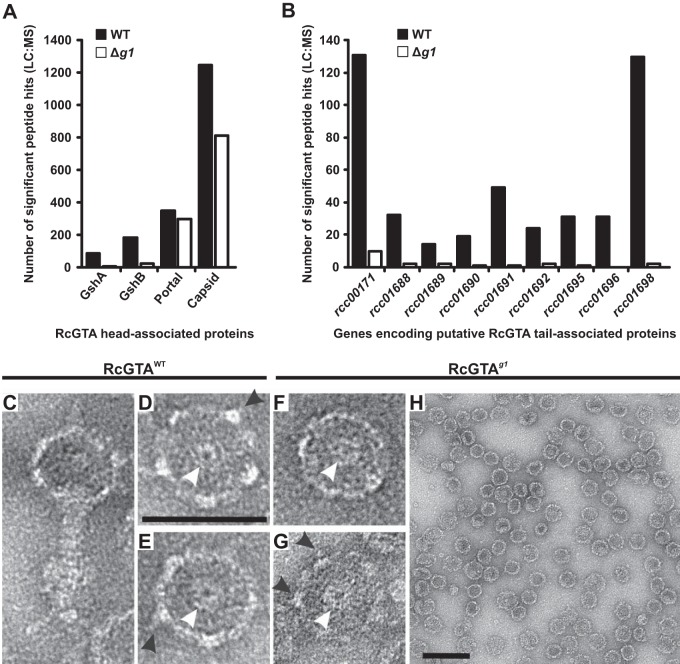
Gp1 is a prerequisite for RcGTA assembly. (A and B) Structural proteome of RcGTA particles. LC-MS/MS analysis of affinity-purified RcGTA particles produced by DE442 wild-type (WT) and *g1* deletion (Δ*g1*) strains. RcGTA head proteins and tail proteins are shown separately in panels A and B, respectively. (D to H) Transmission electron micrographs of RcGTA particles. Images in panels D to G were taken at ×68,000 magnification, and the scale bar in panel D represents 50 nm. The panel H image was taken at ×49,000 magnification, and the scale bar represents 100 nm. Black arrowheads indicate head spikes, and white arrows indicate portal apertures.

**FIG 6:**
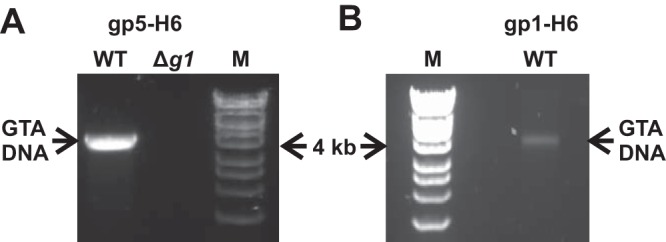
DNA content of affinity-purified RcGTA particles. Agarose gels of DNA extracted directly from chimeric His_6_ (H6)-tagged RcGTAs are shown. His tags were incorporated into nascent RcGTA particles by ectopic expression of His_6_-tagged gp5 (A) or gp1 (B) proteins. Tagged RcGTAs were purified from R. capsulatus DE442 culture supernatants using nickel agarose affinity chromatography. The genotype of the producer cells is indicated directly above each gel, i.e., the wild type (WT) or RcGTA *g1* gene knockout (Δg1). DNA marker HyperLadder 1kb is included for reference (lane M); the locations of the 4-kb reference band and GTA DNA are annotated.

Affinity-purified RcGTA particles were submitted for shotgun liquid chromatography-tandem mass spectrometry (LC-MS/MS) analysis to determine the structural proteome of RcGTA^WT^ versus RcGTA*^g1^*. In terms of number of peptides detected, the two sample types yielded equivalent numbers for the RcGTA capsid and portal proteins (Fig. 5A). The GhsA and GhsB head spike proteins (encoded by *rcc01079* and *rcc01080*, respectively) were represented in both samples; however, 7- to 9-fold-fewer GhsA/B peptides were detected in RcGTA*^g1^* (Fig. 5A). In contrast, peptide hits for the predicted RcGTA tail structures were almost completely absent in the RcGTA*^g1^* samples but abundant for RcGTA^WT^ (Fig. 5B). Transmission electron microscopy images corroborated the proteomic data. RcGTA^WT^ samples yielded intact GTA particles with clearly defined head spikes and portal apertures and dense staining of the heads, possibly indicative of tightly packaged DNA (Fig. 5C to E). RcGTA*^g1^* samples contained no evidence of tail structures, head spikes were present but at reduced frequency, and portal structures were visible (Fig. 5F to H). Overall, RcGTA*^g1^* head structures appeared more prone to damage than the wild type, maturation was often incomplete, and the contrast was poor, probably due to the absence of DNA (Fig. 5F to H). In agreement with data presented earlier (Fig. 3C), DNA extraction from affinity-purified RcGTA^WT^ samples yielded characteristic 4-kb GTA DNA bands, whereas no detectable DNA was recovered from RcGTA*^g1^* samples (Fig. 6A). Similar affinity chromatography using His_6_-tagged gp1 also allowed purification of RcGTA particles from culture supernatant. The overall concentration of RcGTA particles was much lower, presumably because the terminase complex dissociates after packaging is complete, but 4-kb GTA DNA was still recoverable (Fig. 6B). These data demonstrate a direct interaction between gp1 and the broader structural proteome for the first time and support our hypothesis that gp1 is indeed the small terminase.

### RcGTA gp1 binds weakly to DNA.

**FIG 7:**
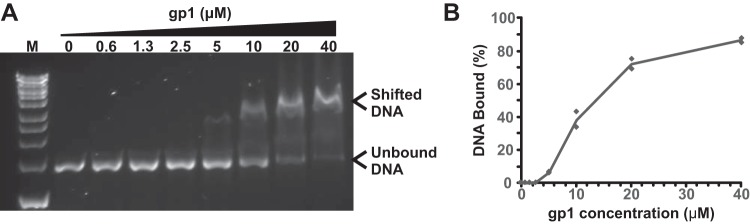
RcGTA gp1 *in vitro* DNA binding. (A) Representative agarose gel (0.8%, wt/vol) showing the stated concentrations of gp1 protein binding to DNA in an electrophoretic mobility shift assay (EMSA). The locations of unbound and shifted DNA are annotated. Substrate DNA in the assay shown is a 1.4-kbp PCR amplification of an arbitrarily chosen region flanking the *rcc01398* gene from R. capsulatus (amplified using *rcc01398* forward and reverse primers [Table 3]). Bioline HyperLadder 1kb DNA marker is shown for size comparison (lane M). (B) Quantification of EMSAs by band intensity analysis. Data shown are the average results of two EMSAs carried out independently in time and with different DNA substrates (flanking the *rcc01397* and *rcc01398* genes). Individual data points are plotted as well as the mean line.

A core role of phage small terminases is to recognize the phage genome and to target it for packaging into preformed capsids. RcGTAs do not package specific DNA, but the large terminase still needs to be recruited to the host genomic DNA to initiate packaging, and it is plausible that this may be achieved via a nonspecific affinity for DNA. In an electrophoretic motility shift assay (EMSA), we tested the ability of RcGTA gp1 to bind DNA *in vitro*. To obtain a high concentration soluble protein, an N-terminal MBP tag was used for gp1 purification. Purified gp1 exhibited low affinity for DNA, with incomplete shifts occurring at micromolar concentrations; 87% of DNA substrate was bound at a 40 μM protein concentration (Fig. 7). Six EMSA DNA substrates were used (351- to 2,944-bp PCR amplicons from six distinct locations in the R. capsulatus genome); however, the identity of the DNA did not substantially affect the binding affinity. The size of the observed shift in DNA mobility was ∼1,500 bp or equivalent to ~975 kDa, which is greater than would be expected for binding of a single protein monomer. The large reduction in mobility of the gp1-DNA complex indicates that gp1 could be binding as an oligomer (small terminases usually form characteristic ring structures), there could be multiple occupancy due to the lack of a specific binding site, and/or the conformation of the DNA may have been altered.

### GTA small terminases can be predicted in other species.

**FIG 8:**
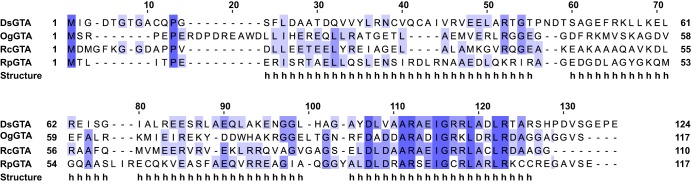
GTA TerS protein alignment. COBALT multiple sequence alignment of putative GTA small terminases from *Rhodobacterales* species Dinoroseobacter shibae (DsGTA), Oceanicola granulosus (OgGTA), Rhodobacter capsulatus (RcGTA), and Ruegeria pomeroyi (RpGTA). The intensity of color for each amino acid is based on the percentage identity. The predicted secondary structure is indicated below the alignment, with “h” indicating helical.

Identification of the RcGTA small terminase allowed us to predict GTA *terS* genes in other alphaproteobacterial species (Table 1), including two previously unannotated ORFs in Parvularcula bermudensis and Dinoroseobacter shibae. Interestingly, we were also able to predict small terminase genes in the distantly related deltaproteobacterium Desulfovibrio desulfuricans and the archaeon Methanoccus voltae (Table 1). In each case, the small terminase gene was immediately upstream of the cognate large terminase gene, the coding sequence for each small terminase was ∼10% to 50% shorter than comparable phage counterparts (Table 1), and the predicted protein structures were almost entirely helical. Overall, the primary amino acid sequences of the various small terminases is poorly conserved, even between those found in closely related species (Fig. 8). However, for the *Rhodobacterales* GTA TerS proteins, there is clear sequence similarity localized at the C termini, specifically the third α-helix (Fig. 8). Conservation of this region supports our findings that the C-terminal helix is required for interaction with TerL and that this interaction constrains TerS sequence divergence.

**TABLE 1:** Predicted small terminases from known GTAs

Host species or phage small terminase^*a*^	Gene name^*b*^	Protein or genome accession no.^*c*^	Size (kDa)	Size (aa)^*d*^
Rhodobacter capsulatus	*rcc001682*	ADE85427	11.5	107
Oceanicola granulosus	*OG2516_RS04255*	EAR49554	12.9	114
Ruegeria pomeroyi	*SPO2267*	AAV95531	12.6	114
Parvularcula bermudensis	NA	CP002156 (1595455–1595796)	13.1	114
*Oceanicaulis alexandrii*	*OA2633_14800*	EAP88801	10.1	92
Methanococcus voltae	*Mvol_0412*	ADI36072	14.6	125
Desulfovibrio desulfuricans	*Ddes_0720*	ACL48628	13.5	125
Bartonella grahamii	*Bgr_16770*	WP_041581600	11.9	107
*Bartonella australis*	*BAnh1_10950*	AGF74963	11.7	109
Dinoroseobacter shibae	NA	CP000830 (2306070–2306432)	12.9	121
*Aeromonas* phage 44RR	Gene 16	NP_932507	17.3	154
Enterobacterial phage T4	Gene 16	NP_049775	18.4	164
*Bacillus* phage SF6	Gene 1	CAK29441	16.0	145
*Bacillus* phage SPP1	Gene 1	CAA39536	20.8	184

a Entries in the last four rows are well-characterized phage small terminases included for comparison.

b NA, not available.

c Where no gene/protein has previously been annotated, the accession number for the bacterial genome is provided with the nucleotide positions of the new ORF indicated in parentheses.

d aa, amino acids.

## DISCUSSION

Gene transfer agents (GTAs) clearly share many structural and mechanistic features with bacteriophages; however, the most striking difference is that GTAs package and transfer random fragments of host DNA without any preference for their own genome. In bacteriophages, DNA packaging is carried out by the terminase complex, which is composed of multimeric small and large terminase subunits. Interestingly, the enterobacterial phage T4 large terminase can promiscuously package heterologous linear DNA fragments into an empty phage head *in vitro* when TerS is absent, reminiscent of GTA-type DNA packaging, but the presence of TerS is essential for terminase activity *in vivo* (46). The large terminase has all the enzymatic capabilities required to package DNA, i.e., a nuclease domain to create free DNA ends at the beginning/end of packaging and an ATPase domain to act as a motor to feed the DNA into the capsid (27, 31, 47). These data demonstrate that TerS is not strictly required for the process of packaging DNA into the capsid but is instead crucial for regulation (28). Depending on the particular phage, TerS forms an oligomeric ring consisting of 8 to 11 identical protein subunits, with the DNA-binding domains arranged around the exterior surface. The TerS ring recognizes the packaging signal in the phage genome and has been proposed to wrap ∼100 bp of DNA around the outside, along the circular surface formed by the DNA-binding domains (48). TerS recruits TerL to make the initial DNA double-strand break but inhibits further DNA cleavage to prevent damage to the phage genome. The TerS/TerL complex docks to the phage head via the oligomeric portal protein, which contains a narrow aperture for the DNA to be fed through. TerS stimulates TerL ATP hydrolysis, and translocation of the packaging complex along the phage genome. Once the genome has been tightly packaged into the capsid, TerL cleaves the DNA again to complete the process. The terminase disassociates, the portal aperture is plugged, and tail assemblies are attached.

Given the role that small terminases play in phage DNA specificity, it is likely that a comparable protein is responsible for random DNA packaging by GTAs; however, no GTA TerS proteins have so far been identified. Taken together, the molecular, genetic, proteomic, and imaging data presented here all support the hypothesis that RcGTA gp1 is the small terminase. RcGTA gp1 is essential for RcGTA gene transfer and DNA packaging (Fig. 3A to C). RcGTA gp1 is predicted to have structural characteristics in common with phage TerS proteins, in particular, a putative coiled-coil domain that is important for oligomerization in phage (Fig. 1) and a conserved C-terminal large terminase interaction domain (Fig. 3D and E and Fig. 8). Analysis of the R. capsulatus DE442 Δ*g1* mutant also allows us to postulate a model to describe RcGTA assembly. RcGTA capsid formation and incorporation of the portal aperture occur independently of the terminase and DNA packaging (Fig. 5C to H). Proteomic analysis of the stalled RcGTA*^g1^* particles did not detect a substantial presence of the large terminase protein (Fig. 5A and B), which suggests that either gp1 recruits TerL to the DNA first and the terminase heterocomplex then recruits the preformed capsids or that the interaction between TerL and the portal is labile in the absence of TerS. Once the terminase-portal-capsid complex is assembled, headful DNA packaging can begin. In the absence of DNA packaging, efficient maturation of the RcGTA heads is impaired and RcGTA production stalls before the tail appendage is attached (Fig. 5).

A crucial difference between the RcGTA small terminase and its phage counterparts is the apparent lack of an N-terminal DNA-binding domain (Fig. 1). Previous work showed that deletion of the N-terminal region of bacteriophage SF6 and SPP1 TerS proteins led to a significant reduction in DNA binding affinity *in vitro* (49), but some binding was still retained. In addition, both T4 and P22 TerS proteins have N- and C-terminal DNA binding activities, with nonspecific DNA binding dependent upon a 9-residue region in the P22 C terminus, R143-K151 (48, 50). Here, we show that the RcGTA TerS protein can bind nonspecifically to DNA at micromolar concentrations (Fig. 7). Absence of the specific DNA-binding domain but retention of nonspecific DNA binding could provide an explanation for random DNA packaging by GTAs. It is possible that the RcGTA TerS protein is also able to bind specific DNA sequences; however, this could not be tested, because we have no evidence to suggest that this occurs *in vivo* and no GTA binding sites are currently known.

In summary, RcGTA gp1 is the first GTA small terminase to be described to date. We hypothesize that GTA small terminases possess all of the regulatory abilities of phage small terminases, but lack of an N-terminal DNA-binding domain abolishes DNA sequence specificity. Loss of the specific DNA binding region could allow nonspecific binding of random DNA sequences, which is the defining characteristic of GTA-type TerS proteins. The greatest barrier to novel GTA identification and an understanding of their true prevalence in the environment is the lack of an effective identification method. Based on the data gained from RcGTA, we were able to predict the small terminases from other known GTAs by using gene size, neighborhood, and protein secondary-structure prediction analyses. Confirmation and in-depth characterization of these proteins could allow us to pinpoint the defining characteristics of GTA-type terminases with a view to enhanced discovery of novel GTAs in existing genome data sets. Furthermore, we also anticipate that the smaller size and simpler organization of GTAs, compared with phages, will provide the opportunity to develop a superior model system for structural and mechanistic studies.

## MATERIALS AND METHODS

### Bacterial strains.

Two wild-type *Rhodobacter* strains were used, rifampin-resistant SB1003 (ATCC BAA-309) and rifampin-sensitive B10 (51). The RcGTA overproducer strain DE442 is of uncertain provenance but has been used in a number of RcGTA reports (44, 52). The E. coli S17-1 strain, which contains chromosomally integrated *tra* genes, was used as a donor for all conjugations. NEB 10-beta competent E. coli (New England Biolabs, NEB) cells were used for standard cloning and plasmid maintenance; T7 Express competent E. coli (NEB) cells were used for overexpression of proteins for purification.

### Cloning.

All cloning reactions were carried out with either the In-Fusion cloning kit (ClonTech) or NEBuilder (NEB) to produce the constructs listed in Table 2. All oligonucleotides were obtained from IDT (Table 3) and designed with an optimal annealing temperature of 60°C when used with Q5 DNA polymerase (NEB). In summary, destination plasmids were linearized using a single restriction enzyme (pCM66T [BamHI], pEHisTEV [NcoI], pKT25 [BamHI], pUT18C [BamHI]) or linearized by PCR (pETFPP_2 using primers CleF and CleR). Inserts were amplified using primers with 15-bp 5′ overhangs that have sequence complementary to the DNA with which it is to be recombined.

**TABLE 2:** Plasmids used in this study

Name	Description^*a*^	Reference or source
pCM66T	Gift from Mary Lidstrom; broad-host-range vector; ColE1, OriV, IncP/TraJ, Kan^r^	Addgene plasmid 74738
pUT18C	Bacterial two-hybrid vector	41
pKT25	Bacterial two-hybrid vector	41
pEHisTEV	Expression vector; T7 promoter, His_6_ tag, TEV cleavage site, Kan^r^	63
pETFPP_22	Expression vector; T7 promoter, His_6_/MBP tags, 3c cleavage site, Kan^r^	64
pCMF170	RcGTA promoter fused to RcGTA *g1* in pCM66T	This study
pJXL1	RcGTA promoter fused to RcGTA *g1*Δh1 in pCM66T	This study
pJXL2	RcGTA promoter fused to RcGTA *g1*Δh3 in pCM66T	This study
pCMF143	T25 fused to RcGTA *g1* in pKT25	This study
pJXL3	T25 fused to RcGTA *g1*Δh1 in pKT25	This study
pJXL4	T25 fused to RcGTA *g1*Δh3 in pKT25	This study
pCMF144	T18 fused to RcGTA *g2* in pUT18C	This study
pCMF238	T18 fused to RcGTA *g2* ATPase domain in pUT18C	This study
pCMF239	T18 fused to RcGTA *g2* nuclease domain in pUT18C	This study
pCMF153	His_6_-RcGTA *g1* in pEHisTEV	This study
pCMF166	His_6_-MBP-RcGTA *g1* in pETFPP_22	This study
pCMF142	RcGTA promoter fused to RcGTA *g5*-His_6_ in pCM66T	This study
pCMF173	RcGTA promoter fused to RcGTA *g1*-His_6_ in pCM66T	This study
pCMF172	RcGTA *g1* flanking DNA interrupted with Gent^r^ in pCM66T	This study

a Kan, kanamycin; Gent, gentamicin.

**TABLE 3:** Oligonucleotides used in this study

Name	Sequence (5′–3′)
pGTA F^1^^*a*^	CGACTCTAGAGGATCGATTGTCGATCAGATCAC
pGTA R^1^^*a*^	GCTGACCATCGCCAGGGCCAGTTCC
*g1* (66T) R	CGGTACCCGGGGATCTCAACCTCCTGCGGCGTC
pGTA *g1* Δh1 inv F	CAAGACATGAAAGGGGTTCGCCAG
pGTA *g1* Δh1 inv R	CCCTTTCATGTCTTGCGTGACCCG
pGTA *g1* Δh3 R	CGGTACCCGGGGATCCTAACCGGCAACTTGTCTGC
T25-*g1* F	CGACTCTAGAGGATCTGAAAGGGGTTCGCCAG
T25-*g1* R	AGGTACCCGGGGATCTCAACCTCCTGCGGCGTC
T25-*g1* Δh1 F	CGACTCTAGAGGATCTGGACATGGGGTTCAAG
T25-*g1* Δh3 R	AGGTACCCGGGGATCCTAACCGGCAACTTGTCTGC
T18C-*g2* F	CGACTCTAGAGGATCTGGGGGGGCTTGGGAACAAT
T18C-*g2* R	CGGTACCCGGGGATCTCAAAGCCCGCGCACCTG
T18C-*g2* Nuc F	CGACTCTAGAGGATCGTATGGTTCTGCTGGAGGATGTC
T18C-*g2* ATP R	CGGTACCCGGGGATCCTAGACATCCTCCAGCAGAAC
H6-*g1* F^2^^*a*^	TTTCAGGGCGCCATGGACATGGGGTTCAAG
H6-*g1* R^2^^*a*^	CCGATATCAGCCATGTCAACCTCCTGCGGCGTC
MBP-*g1* F	TCCAGGGACCAGCAATGGACATGGGGTTCAAG
MBP-*g1* R	TGAGGAGAAGGCGCGGTCAACCTCCTGCGGCGTC
*g1*-H6 R	CGGTACCCGGGGATCTCAATGGTGATGGTGATGGTGACCTCCTGCGGCGTCGCG
*g5*-H6 F	CTGGCGATGGTCAGCATGAAGACCGAGACCAAG
*g5*-H6 R	CGGTACCCGGGGATCTTAGTGATGGTGATGGTGATGCGAGGCGGCAAACTTCAAC
*g1* UP R	GGGAATCAGGGGATCCTGGCGAACCCCTTTCAT
*g1* DOWN F	AACAATTCGTTCAAGAGACAAGTTGCCGGTGTCG
*g1* DOWN R	CGGTACCCGGGGATCGTCCAAATACGCCCTTGCG
Gent F	GATCCCCTGATTCCCTTTGT
Gent R	CTTGAACGAATTGTTAGG
*rcc01397* F^3^^*a*^	CGACTCTAGAGGATCCCAGCGCGTAGATCGACG
*rcc01397* R^3^^*a*^	CGGTACCCGGGGATCGCGATTGCCAACATCGCC
*rcc01398* F^4^^*a*^	CGACTCTAGAGGATCCGCTTTCGCCTGCGCCTGC
*rcc01398* R^4^^*a*^	CGGTACCCGGGGATCCTCGGCATGGATCCAGTGC
*gafA* F^5^^*a*^	CGACTCTAGAGGATCAGGAAGCCCTTGCCATAGG
*gafA* R^5^^*a*^	CGGTACCCGGGGATCGCGAAGCTGGAGTTCAACC
*rcc00555* F^6^^*a*^	TAATCGCGGCCTCGAATCGTCATCGACCTGAAGGC
*rcc00555* R^6^^*a*^	ATTTTGAGACACAACCGAAATCAGGTTAACGATCC

a Primers used to generate EMSA substrate DNA; F and R followed by the same superscript number (1 to 6) indicate a primer pair.

### Transformation.

Plasmids were introduced into E. coli by standard heat shock transformation (53) and into *Rhodobacter* by conjugation. For conjugation, 1-ml aliquots of an E. coli S17-1 donor containing the plasmid of interest and the *Rhodobacter* recipient were centrifuged at 5,000 × *g* for 1 min, washed with 1 ml SM buffer, centrifuged again, and resuspended in 100 μl SM buffer. Ten microliters of concentrated donor and recipient cells were mixed and spotted onto YPS agar or spotted individually as negative controls. Plates were incubated overnight at 30°C. Spots were scraped, suspended in 100 μl YPS broth, and plated on YPS plus 100 μg ml^−1^ rifampin (counterselection against E. coli) plus 10 μg ml^−1^ kanamycin (plasmid selection). Plates were incubated overnight at 30°C and then restreaked onto fresh agar to obtain single colonies.

### Gene knockouts.

Knockouts were created by RcGTA transfer. pCM66T plasmid constructs were created with a gentamicin resistance cassette flanked by 500 to 1,000 bp of DNA from either side of the target gene. Assembly was achieved by a one-step, four-component NEBuilder (NEB) reaction and transformation into NEB 10-beta cells. Deletion constructs were introduced into the RcGTA hyperproducer strain by conjugation, and a standard GTA bioassay was carried out to replace the intact chromosomal gene with the deleted version.

### *Rhodobacter* gene transfer assays.

In *Rhodobacter*, the assays were carried out essentially as defined by Leung and Beatty (54). RcGTA donor cultures were grown anaerobically with illumination in YPS for ∼48 h, and recipient cultures were grown aerobically in RCV for ∼24 h. For overexpression experiments, donor cultures were first grown aerobically to stationary phase and then anaerobically for 24 h. Cells were cleared from donor cultures by centrifugation, and the supernatant was filtered through a 0.45-μm syringe filter. Recipient cells were concentrated 3-fold by centrifugation at 5,000 × *g* for 5 min and resuspension in one-third volume G-buffer (10 mM Tris-HCl [pH 7.8], 1 mM MgCl_2_, 1 mM CaCl_2_, 1 mM NaCl, 0.5 mg ml^−1^ bovine serum albumin [BSA]). Reactions were carried out in polystyrene culture tubes (Starlab) containing 400 μl G-buffer, 100 μl recipient cells, and 100 μl filtered donor supernatant and then incubated at 30°C for 1 h. A 900-μl volume of YPS was added to each tube and incubated for a further 3 h. Cells were harvested by centrifugation at 5,000 × *g* and plated on YPS plus 100 μg ml^−1^ rifampin (for standard GTA assays) or 3 μg ml^−1^ gentamicin (for gene knockouts).

### DNA purification.

To isolate total intracellular DNA, 1-ml samples of relevant bacterial cultures were taken for each nucleic acid purification replicate. Generally, sampling occurred during stationary phase, but for overexpression experiments, samples were taken 6 h and 24 h after transition to anaerobic growth. Total DNA was purified according to the “Purification of Nucleic Acids by Extraction with Phenol:Chloroform” protocol described previously (53). To isolate extracellular DNA contained in RcGTA virions, R. capsulatus DE442 cultures (23 ml) were grown anaerobically with illumination in YPS for ∼48 h at 30°C. Cells were cleared from the cultures by centrifugation at 15,000 × *g* for 10 min, and the supernatant was filtered through a 0.45-μm syringe filter. RcGTAs were precipitated by the addition of polyethylene glycol 8000 to a final concentration of 10% (wt/vol) and then incubated at 4°C for 1 h with continuous rolling. Precipitated RcGTAs were pelleted by centrifugation at 10,000 × *g* for 10 min. The pellet was resuspended in 500 μl G-buffer. Bacterial DNA and RNA were removed by overnight incubation with BaseMuncher nuclease (Expedeon) in the presence of 10 mM MgCl_2_ at 30°C. Nuclease digestion was inhibited by the addition of 50 mM EDTA. DNA was extracted with phenol-chloroform-isoamyl alcohol (25:24:1, pH 8.0) as previously described (53).

### Bacterial two-hybrid assays.

The bacterial two-hybrid assay procedure and resources were as described previously (41). Plasmids encoding T18 (pUT18C and derivatives) and the compatible plasmids encoding T25 (pKT25 and derivatives) were introduced pairwise into competent BTH101 by cotransformation. Selection was done using LB agar containing 50 μg/ml kanamycin, 100 μg/ml ampicillin, 1 mM IPTG (isopropyl-β-d-thiogalactopyranoside) and 80 μg/ml X-Gal (5-bromo-4-chloro-3-indolyl-β-d-galactopyranoside), and plates were incubated at 30°C for 24 to 48 h. The phenotype of BTH101 (Cya^–^) can be complemented if the two domains of adenylate cyclase (T18 and T25) are brought into close proximity, and this can be achieved by fusing interacting protein partners to each domain. The readout for complementation of the Cya^–^ phenotype (indicating a positive interaction between the two fusion proteins) is the induction of *lac* (blue colonies on IPTG and XGal), whereas no induction (white colonies) indicates no fusion protein interaction.

### Assay of β-galactosidase activity.

Colonies obtained from the bacterial two-hybrid assay plasmids introduced into BH101 were spotted onto selective agar. The confluent spots were used to inoculate 200-μl aliquots of LB supplemented with 50 μg/ml kanamycin, 100 μg/ml ampicillin, and 1 mM IPTG in a 96-well plate. Plates were covered and incubated for 16 h at 30°C with agitation. Absorbance (optical density at 600 nm [OD_600_]) readings were taken using a plate reader. In a second 96-well plate, 80-μl aliquots of permeabilization solution (100 mM Na_2_HPO_4_, 20 mM KCl, 2 mM MgSO_4_, 0.06% [wt/vol] CTAB [cetyltrimethylammonium bromide], 0.04% [wt/vol] sodium deoxycholate, 0.0054% [vol/vol] TCEP Tris(2-carboxyethyl)phosphine hydrochloride]) were prepared. Twenty-microliter aliquots from each well of the cultured bacteria were added to the corresponding wells of the plate containing the permeabilization solution, and the mixtures were incubated at room temperature for 15 min. Twenty-five microliters of the permeabilized samples was then added to 150 μl of substrate solution (60 mM Na_2_HPO_4_, 40 mM NaH_2_PO_4_, 1 mg/ml ONPG [*o*-nitrophenyl-β-d-galactopyranoside], and 0.0027% [vol/vol] TCEP) that had been placed in a third 96-well plate. Absorbance (OD_420_) readings were taken in the plate reader at 10-min intervals over 60 min at 30°C. The maximum 2-point slope was calculated (ΔOD_420_/min/ml).

### Affinity purification of RcGTA particles.

Purification of RcGTA particles was carried out as previously described, with minor modifications (15, 45). Plasmids pCMF142 and pCMF173 (Table 2) were conjugated into the RcGTA overproducer strain R. capsulatus DE442 and an isogenic RcGTA *g1* deletion strain. pCMF142 and pCMF173 use the RcGTA promoter to express the RcGTA major capsid protein or gp1, respectively, with a hexahistidine purification tag incorporated at the C terminus. One-hundred-milliliter cultures of DE442 and DE442 Δ*g1* were grown in YPS medium to stationary phase at 30°C, anaerobically with constant illumination. The cultures were cleared by centrifugation at 15,000 × *g* for 10 min, followed by syringe filtration through a 0.45-μm pore filter. Tris-HCl (pH 8) was added to a final concentration of 10 mM. Each filtrate was mixed with 3 ml Amintra Ni-agarose beads (Expedeon) preequilibrated with G* buffer (10 mM Tris-HCl [pH 8], 1 mM MgCl_2_, 1 mM CaCl_2_, and 1 mM NaCl) and then incubated at room temperature for 1 h with agitation. Beads were applied to 25-ml gravity flow columns (Thermo Fisher) and washed with 200 ml G* buffer supplemented with 40 mM imidazole. RcGTA particles were eluted using 5 ml G* buffer supplemented with 400 mM imidazole. Eluted RcGTAs were concentrated and imidazole depleted to <1 mM by iterative dilution and ultrafiltration using a 100-kDa Spin-X UF20 device (Corning).

### Electron microscopy.

Affinity-purified RcGTAs were directly applied to 200-mesh copper grids with a Formvar/carbon support film and allowed to adsorb for 4 min. The grids were washed with 3 drops of deionized water and then negatively stained with uranyl acetate solution (55). Samples were analyzed on a Tecnai 12 BioTWIN G2 transmission electron microscope operating at 120 kV, and images were captured using a Ceta camera (Thermo Fisher).

### Protein purification.

Protein overexpression was carried out in the NEB Express E. coli strain (NEB) containing the relevant T7 expression plasmid (Table 2). Expression from the T7 promoter was induced at mid-exponential growth phase with 0.2 mM IPTG at 20°C overnight. His_6_-tagged (56) and MBP-tagged (40) proteins were purified as described previously. All chromatography steps were carried out on an AKTA Prime instrument (GE Healthcare). Purified proteins were concentrated in a Spin-X UF centrifugal concentrator (Corning). Samples were stored at –80°C in binding buffer plus 50% glycerol.

### EMSA.

DNA substrates were prepared by PCR amplification with oligonucleotides indicated in Table 3 and cleaned with a Monarch DNA cleanup kit (NEB). Ten-microliter electrophoretic mobility shift assay (EMSA) mixtures contained 100 ng of DNA, binding buffer based on reference 57 [25 mM HEPES, 50 mM K-glutamate, 1 mM dithiothreitol, 0.05% Triton X-100, 4% glycerol, 1 μg poly(dI-dC), pH 8.0], and purified protein at stated concentrations. Binding assays were carried out at room temperature for 30 min. Samples were run on a 0.8% agarose gel in 0.5× Tris-borate-EDTA (TBE) at 80 V for 2 h at room temperature. Gels were stained with SYBR Safe (Invitrogen) and imaged on a GelDoc transilluminator (Bio-Rad).

### Sample preparation for mass spectrometry.

For MALDI-MS/MS protein identification, purified RcGTA samples were run on a TEO-Tricine 4–12% SDS minigel (Expedeon) at 150 V for 45 min. Gels were stained with InstantBlue protein stain (Expedeon) for 1 h before destaining with ultrapure water for 1 h. Protein bands of interest were excised. For shotgun LC-MS/MS, samples were run on a 7-cm NuPAGE Novex 10% bis-Tris gel (Life Technologies) at 200 V for 6 min. Gels were stained with SafeBLUE protein stain (NBS biologicals) for 1 h before destaining with ultrapure water for 1 h.

In-gel tryptic digestion was performed after reduction with dithioerythritol and *S*-carbamidomethylation with iodoacetamide. Gel pieces were washed two times with 50% (vol/vol) aqueous acetonitrile containing 25 mM ammonium bicarbonate and then once with acetonitrile and then dried in a vacuum concentrator for 20 min. Sequencing-grade modified porcine trypsin (Promega) was dissolved in the 50 mM acetic acid supplied by the manufacturer and then diluted 5-fold by adding 25 mM ammonium bicarbonate to give a final trypsin concentration of 0.02 μg/μl. Gel pieces were rehydrated by adding 10 μl of trypsin solution, and after 5 min, enough 25 mM ammonium bicarbonate solution was added to cover the gel pieces. Digests were incubated overnight at 37°C.

### MALDI-MS/MS.

A 1-μl aliquot of each peptide mixture was applied to a ground steel MALDI target plate, followed immediately by an equal volume of a freshly prepared 5-mg/ml solution of 4-hydroxy-α-cyano-cinnamic acid (Sigma) in 50% aqueous (vol/vol) acetonitrile containing 0.1% (vol/vol) trifluoroacetic acid.

Positive-ion MALDI mass spectra were obtained using a Bruker Ultraflex III in reflectron mode, equipped with a Nd:YAG smart beam laser. MS spectra were acquired over a range of 800 to 4,000 *m/z*. Final mass spectra were externally calibrated against an adjacent spot containing 6 peptides (des-Arg1-bradykinin, 904.681; angiotensin I, 1,296.685; Glu1-fibrinopeptide B, 1,750.677; ACTH [1-17 clip], 2,093.086; ACTH [18-39 clip], 2,465.198; ACTH [7-38 clip], 3,657.929.). Monoisotopic masses were obtained using a SNAP averaging algorithm (C 4.9384, N 1.3577, O 1.4773, S 0.0417, H 7.7583) and a signal-to-noise (S/N) threshold of 2.

For each spot, the 10 strongest precursors, with a S/N ratio greater than 30, were selected for MS/MS fragmentation. Fragmentation was performed in LIFT mode without the introduction of a collision gas. The default calibration was used for MS/MS spectra, which were baseline subtracted and smoothed (Savitsky-Golay filter; width, 0.15 *m/z*; cycles, 4); monoisotopic peak detection used a SNAP averaging algorithm (C 4.9384, N 1.3577, O 1.4773, S 0.0417, H 7.7583) with a minimum S/N ratio of 6. Bruker FlexAnalysis software (version 3.3) was used to perform spectral processing and peak list generation.

### Shotgun LC-MS/MS.

Peptides were extracted by washing three times with 50% (vol/vol) aqueous acetonitrile containing 0.1% trifluoroacetic acid (vol/vol), before being dried in a vacuum concentrator and reconstituted in aqueous 0.1% trifluoroacetic acid (vol/vol). Samples were loaded onto a nanoAcquity ultraperformance liquid chromatography (UPLC) system (Waters) equipped with a nanoAcquity Symmetry C_18_, 5-μm trap (180 μm by 20 mm; Waters) and a nanoAcquity HSS T3 1.8-μm C_18_ capillary column (75 μm by 250 mm; Waters). The trap wash solvent was 0.1% (vol/vol) aqueous formic acid, and the trapping flow rate was 10 μl/min. The trap was washed for 5 min before switching the flow to the capillary column. Separation used a gradient elution of two solvents (solvent A, aqueous 0.1% [vol/vol] formic acid; solvent B, acetonitrile containing 0.1% [vol/vol] formic acid). The capillary column flow rate was 350 nl/min, and the column temperature was 60°C. The gradient profile was linear from 2% to 35% B over 20 min. All runs then proceeded to wash with 95% solvent B for 2.5 min. The column was returned to initial conditions and reequilibrated for 25 min before subsequent injections.

The nanoLC system was interfaced with a maXis HD LC-MS/MS system (Bruker Daltonics) with a CaptiveSpray ionization source (Bruker Daltonics). Positive electrospray ionization (ESI)-MS and MS/MS spectra were acquired using the AutoMS/MS mode. Instrument control, data acquisition, and processing were performed using Compass 1.7 software (microTOF control, Hystar, and DataAnalysis, Bruker Daltonics). Instrument settings were as follows: ion spray voltage, 1,450 V; dry gas, 3 liters/min; dry gas temperature, 150°C; ion acquisition range, 150 to 2,000 *m/z*; MS spectra rate, 2 Hz; MS/MS spectra rate, 1 Hz at 2,500 counts to 10 Hz at 250,000 counts; cycle time, 3 s; quadrupole low mass, 300 *m/z*; collision radio frequency (RF), 1,400 V (peak-to-peak); transfer time, 120 ms. The collision energy and isolation width settings were automatically calculated using the AutoMS/MS fragmentation table, an absolute threshold of 200 counts, and preferred charge states of 2 to 4, singly charged ions excluded. A single MS/MS spectrum was acquired for each precursor, and former target ions were excluded for 0.8 min unless the precursor intensity increased 4-fold.

### Bioinformatics.

Tandem mass spectral data were submitted to a database search against the unrestricted NCBI nr database (version 20190131; 187,087,713 sequences and 68,237,485,887 residues) using a locally running copy of the Mascot program (Matrix Science Ltd., version 2.5.1), through the Bruker ProteinScape interface (version 2.1). The search criteria specified were as follows: enzyme; trypsin; fixed modifications, carbamidomethyl (C); variable modifications, oxidation (M); peptide tolerance, 150 ppm; MS/MS tolerance, 0.75 Da; instrument used, MALDI-TOF-TOF. Results were filtered to accept only peptides with an expect score of 0.05 or lower.

Helix-turn-helix predictions were carried out using NPS@ (37, 38) and Gym2.0 (39) with the default settings. Protein secondary-structure and coiled-coil predictions were made using JPRED4 (34) and COILS (35), respectively. Protein three-dimensional (3D) structures were predicted using RaptorX (36). Molecular graphics/analyses were performed with the UCSF Chimera package v1.13 (58). Chimera is developed by the Resource for Biocomputing, Visualization, and Informatics at the University of California, San Francisco (supported by NIGMS P41-GM103311). COBALT was used for protein sequence alignments, and PROMALS3D was used for alignment of predicted protein structure (59, 60). Jalview was used to visualize alignments (61). FIJI software was used for image analysis (62). Figure graphics were produced using CorelDraw 2018. Statistical analysis was carried out using SigmaPlot software version 13 (Systat Software, Inc.), and, for each use, the test parameters are indicated in the text and/or figure legends.
